# Giant cell tumour of bone: new treatments in development

**DOI:** 10.1007/s12094-014-1268-5

**Published:** 2015-01-24

**Authors:** A. López-Pousa, J. Martín Broto, T. Garrido, J. Vázquez

**Affiliations:** 1 Hospital de la Santa Creu i Sant Pau, Barcelona, Spain; 2 Hospital Universitari Son Espases, Palma de Mallorca, Spain; 3 Amgen S.A., Barcelona, Spain; 4 Medical Oncology Department, Hospital de la Santa Creu i Sant Pau, Autonomous University of Barcelona, Mas Casanovas 90, ES-08041 Barcelona, Spain

**Keywords:** Giant cell tumour of bone, Pathophysiology, Surgery, Denosumab

## Abstract

Giant cell tumour of bone (GCTB) is a benign osteolytic tumour with three main cellular components: multinucleated osteoclast-like giant cells, mononuclear spindle-like stromal cells (the main neoplastic components) and mononuclear cells of the monocyte/macrophage lineage. The giant cells overexpress a key mediator in osteoclastogenesis: the RANK receptor, which is stimulated in turn by the cytokine RANKL, which is secreted by the stromal cells. The RANK/RANKL interaction is predominantly responsible for the extensive bone resorption by the tumour. Historically, standard treatment was substantial surgical resection, with or without adjuvant therapy, with recurrence rates of 20–56 %. Studies with denosumab, a monoclonal antibody that specifically binds to RANKL, resulted in dramatic treatment responses, which led to its approval by the United States Food and Drugs Administration (US FDA). Recent advances in the understanding of GCTB pathogenesis are essential to develop new treatments for this locally destructive primary bone tumour.

## Introduction: new insights into pathophysiology

### Definition, epidemiology and natural course

Giant cell tumour of bone (GCTB) is a rare osteolytic tumour that is responsible for approximately 6 % of all primary bone tumours. Reported annual incidence ranges between 1 and 6 per 10 million persons [1, 2] to approximately 1 per million in the US, Western Australia, Japan and Sweden [3]. It typically affects adults aged between 20 and 50 years [4–6], with a slightly higher incidence among females (1.7 per 10 million in females versus 1.5 per 10 million in males) [1, 4–6]. GCTB is typically located in the epiphysis of bones, causing localised tenderness and swelling, reduced joint mobility, and pain that is often severe and intractable [6]. It usually develops in long bones but can also occur in unusual locations. The Enneking staging classification, based on radiological, histological and clinical features, is the most commonly used (Table 1) [7]. There is also a radiological grading system established by Campanacci et al. [8] that classifies GCTB into three radiographic types (I, intramedullary lesion confined to bone; II, thinned, expanded cortex, III, cortical breakout), and is roughly comparable with the staging system of Enneking et al. [7]

**Table 1:** Enneking classification of GCTB [7]

Stage	%	Description
Stage I (latent)	15	Confined totally by bone
		Asymptomatic
		Inactive on bone scan
		Histologically benign
Stage II (active)	70	Expanded cortex with no breakthrough
		Symptomatic
		Often have pathological fracture
		Active on bone scan
		Histologically benign
Stage III (aggressive)	15	Rapidly growing mass
		Cortical perforation with soft tissue mass
		May metastasize
		Symptomatic
		Extensive activity on bone scan
		Histologically benign
Malignant	Very rare	Sarcomatous lesion contiguous with benign GCT

Symptoms are variable; some patients may be asymptomatic until they develop a pathologic fracture while others complain of pain at the adjacent joint and limited range of motion. There may also be swelling and even a visible mass, if the tumour has grown for a long time. Other commonly reported symptoms include muscular or nerve pain [6].

The tumour is locally aggressive and destructive, and it grows rapidly, destroying bone and spreading into surrounding soft tissues [9]. If it is surgically resected, there is a substantial probability of recurrence, which seems to be greater in some locations associated with more difficult treatment, such as the distal radius and the proximal femur [10]. In the absence of treatment, the continued and unchecked tumour growth leads to complete destruction of the bone, physical deformity and the possibility of loss of limb.

The most common site of metastasis is the lung, occurring at a frequency of 1–6 % [11, 12]. Pulmonary metastases are usually histologically benign and their course is indolent. The standard of care is surgical resection, and prognosis is generally good. If resection is not possible, they can be left untreated [6, 11, 13].

Rarely, in less than 1 % of cases, GCTB may undergo malignant transformation that is known to result in a poor prognosis for the patient [6, 14]. The malignancy may arise as a result of dedifferentiation of the primary tumour or secondary to radiation therapy (approximately 50 % of cases) [11]. The most commonly observed transformation is to a high-grade sarcoma, usually an osteosarcoma, however, in rare cases this transformation may result in the formation of a fibrosarcoma or a classically denominated malignant fibrous histiocytoma. The mean time after initial GCTB diagnosis to malignant transformation is around 19 years in patients with spontaneous transformation and around 9 years in post-radiation cases [11].

### Pathophysiology

The histopathology of GCTB reveals the presence of marked haemorrhage and three major cell types: multinucleated giant cells, stromal cells and mononuclear cells of the monocyte/macrophage lineage [15, 16].

The spindle-like stromal cells are the main neoplastic components, and appear to be activated by fibroblasts that secrete type I and III collagen and possess parathormone receptors [17, 18]. They promote giant cell formation by expressing and secreting a variety of chemotactic factors (cytokines such as interleukin [IL]-6, IL-8, IL-11, IL-17, IL-34, basic fibroblast growth factor [b-FGF], tumour necrosis factor [TNF]-α, vascular endothelial growth factor [VEGF], macrophage colony-stimulating factor [M-CSF], RANKL, cathepsin K; chemokines such as IL-8, TGF-β1 and stromal derived factor-1 [SDF-1]; and enzymes such as matrix metalloproteinase [MMP]-9 and MMP-13) [19–22]. All these factors serve to engage and differentiate circulating monocytes into macrophages [23]. Of these factors, SDF-1 appears to act as a chemoattractant involved in the recruitment of monocytes [23]. Furthermore, some studies have correlated the expression of VEGF and MMP-9 with the extent of bone destruction and probability of recurrence [24].

**Fig. 1:**
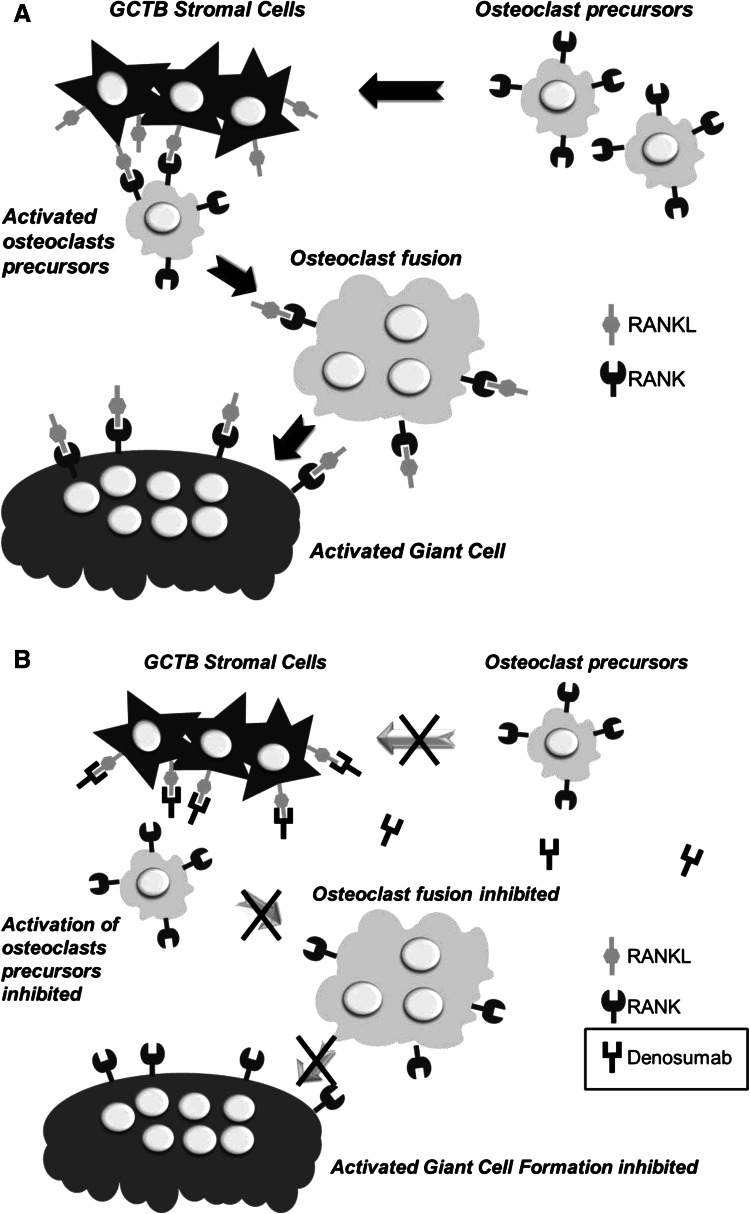
**a** Mechanism of increased bone resorption in GCTB: central role of the RANK/RANKL interaction [33–35]. **b** Proposed mechanism of action of denosumab in GCTB [96]

Giant cells are directly responsible for the increased bone resorption observed within the lesion [25, 26]. They are considered to be reactive macrophages that have acquired osteoclastic activity as a result of their stimulation by stromal cells, which modifies their gene expression pattern within the osseous environment [27]. Giant cells also drive increased expression of a key mediator in osteoclastogenesis: the RANK receptor [28]. Activation of this receptor by RANKL, which is secreted by stromal cells, promotes osteoclast formation, activation, function, and survival [29–32]. Thus, leading to the increased level of bone resorption observed within the GCTB lesion. In addition, the activated osteoclasts, in turn, release tumour growth factors into the bone microenvironment, initiating a tumour/bone vicious cycle (Fig. 1a) [33–35].

**Fig. 2:**
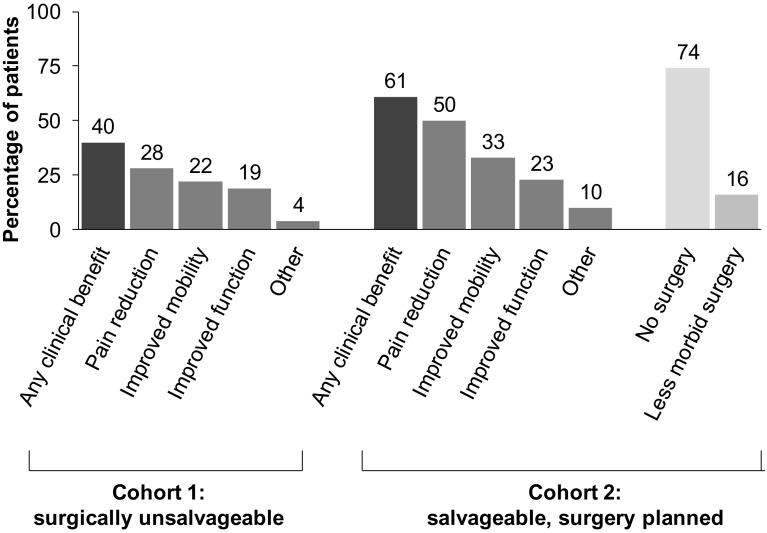
Clinical benefits (investigator-determined) observed with denosumab in patients with primary or recurrent GCTB participating in a phase 2, open-label study [98]

The underlying cause of the increased RANKL expression by stromal cells is unknown, however, this phenomenon is reduced after elimination of the giant cells [32]. Conversely, giant cells are clearly dependent on RANKL signalling by stromal cells [32, 36]. Thus, it is possible that GCTB promotes a pathological variation of the normal physiological interdependence of osteoblast and osteoclast populations in bone [37, 38].

Cytogenetic abnormalities have been observed in up to 72 % of patients with GCTB, yet to date, no uniform aberrations have been identified [15, 39, 40]. Telomeric associations (reductions in telomere length with an average loss of 500 base pairs) are the most frequent chromosomal aberrations and the telomeres most commonly affected are 11p, 13p, 14p, 15p, 19q, 20q and 21p [39, 40].

There is also a hypothesis that the origin of GCTB could be linked to a form of bone injury, as in some cases GCTB appears in locations associated with prior trauma [41, 42]. In this scenario, GCTB could be considered a local reactive condition secondary to a haemorrhage due to bone injury and/or defective collagen in the matrix or in the vessel wall. It is possible that the haemorrhage serves to provide fresh monocytes and plasma proteins that initiate activation of stromal cells, which in turn stimulate conversion of giant cells into active osteoclasts [41]. Once the primary lesion occurs, the stromal cells would be capable of re-forming the tumour in secondary tumour sites or after surgical removal, thanks to their proliferative and tumour-initiating properties [17, 43, 44]. However, it seems that other transformational factors are required since injection of isolated stromal cells into immunocompromised mice does not produce giant cells [45, 46]. Some studies suggest that metastases could result from tumour emboli travelling to distant sites [47, 48].

## Treatment

### Surgical treatment

Surgical removal of the tumour with wide excision or intralesional curettage and placement of cement (polymethyl methacrylate) has been historically the preferred treatment for GCTB [10, 49–52]. The challenge with surgery is to remove as much of the tumour as possible while leaving the joint intact. Wide excision is associated with poor functional outcome and greater surgical complications [53–55]. Therefore, intralesional curettage has been the mainstay of treatment for the majority of patients with stage I or II tumours. Wide excision is usually reserved for more aggressive stage III tumours with extraosseous extension or otherwise unresectable tumours [6, 56, 57]. However, sometimes the tumour is unresectable or surgery is not recommended due to age, patient comorbidities or risk of severe morbidity, such as joint removal or loss of limbs.

Aggressive GCTBs may require wide excision and reconstruction with a modular endoprosthesis; the most commonly used synthetic grafts are made from polymethyl methacrylate (PMMA). These grafts are known to generate an exothermic reaction that increases thermal necrosis of tumour cells and an inflammatory reaction, consequently resulting in an improved patient recovery and tumour removal [58, 59].

The main complications associated with surgery include pathologic fracture and postoperative infection. Postoperative infection occurs in 2–25 % of patients, and its incidence is probably greater with more extensive surgery involving en bloc resection and placement of an endoprosthesis [60–63]; whereas pathologic fracture is associated with an increased rate of recurrence and a poorer functional outcome [64].

There is a recognised tendency for GCTBs to recur locally in many cases following surgery, even in the soft tissues adjacent to the primary bone location [5, 10, 65, 66]. In one of the largest published cohorts, a multicentre retrospective study in 294 Scandinavian patients, Kivioja et al. [52] reported recurrence rates ranging between 20 % for patients with PMMA cementation following intralesional curettage, and 56 % for patients without cementation. In contrast, wide excision is reported to be associated with a lower risk of local recurrence (0–12 %) than intralesional curettage (12–65 %) [5, 67–69]. The use of improved surgical techniques, such as extensive mechanical burr drilling of the tumour wall after curettage or adjuvant cryoablation with liquid nitrogen, has further decreased recurrence rates in some centres, but these techniques have not yet been widely adopted. In the study by Malawer et al. [70] only 2.3 % of patients recurred after primary treatment with cryosurgery, although this percentage increased to 7.9 % when second-line treatments were also considered.

### Chemical adjuvant therapy

Currently, there is no standard or approved first-line medical treatment for GCTB. Surgical treatment may be combined with chemical adjuvant therapies. Some of the treatments commonly applied to the affected area are: alcohols [59, 71, 72], phenol [71, 73], hydrogen peroxide [71, 74, 75], and zinc chloride [76]. Hydrogen peroxide has been found to increase the penetration of phenol into the surrounding tissues [75]. Use of chemical adjuvants has been shown to reduce the percentage of recurrences in some studies [77], although others failed to demonstrate any impact [78]. Furthermore, these adjuvants must be used with caution, to avoid chemical burns.

### Radiation therapy

Radiation therapy has been used to treat GCTB since 1932 [79] and its efficacy has been demonstrated by several studies in patients for whom surgery was not feasible [80, 81]. Specialised techniques such as 3-D conformal radiotherapy (RT) and intensity-modulated radiotherapy (IMRT) have been associated with good local control rates in patients with GCTB in locations that are not accessible by surgical resection [82, 83]. However, some reports have suggested an increased risk of malignant transformation into post-radial sarcoma [84].

The better safety profile of the new drugs available to inhibit osteoclastogenesis has decreased the use of RT in GCTB [85].

### Embolisation and laser photoablation

Embolisation is made by hyperselective catheterisation and embolisation of the arteries that feed the pathological lesion with the most appropriate embolic agent. Typically, Gelfoam, polyvinyl alcohol (PVA) particles, and coils are used for embolisation; other agents include tissue adhesive, ethanol, and microfibrillar collagen. Occlusion of the vessels decreases the volume of the tumour, but multiple procedures are frequently necessary [86]. Photoablation with an argon laser is another therapy that can lead to successful tumour necrosis [87].

Given the high vascularity and morbidity associated with surgical resection and/or radiation therapy, embolisation has been reported to be useful within 24–48 h prior to these therapies [88, 89], to prevent recanalisation. The combined use of preoperative embolisation and adjuvants, including radiation therapy and intraoperative phenol and nitrogen, can decrease local recurrence to less than 10 % [90].

Serial embolisation is also used as primary treatment in some patients with GCTB of the extremities, especially for tumours with large cortical defects or joint involvement and for those with large GCTBs of the sacrum. This procedure has a low morbidity rate and has been shown to be effective in preserving function and relieving pain in selected patients [91–93].

### Drug therapy

#### Denosumab

In parallel with an improved understanding of the pathogenesis of the tumour, other treatment options for GCTB are continuously being explored. The discovery of the involvement of the RANK/RANKL pathway has recently led to the use of the monoclonal antibody denosumab [94]. To date, denosumab is the first and only drug approved by the United States (US) Food and Drug Administration (FDA) and, in Europe, by the European Medicine Agency (EMA) for GCTB [95].

##### Indication

Since June 2013, denosumab is indicated in the US for treatment of adults and skeletally mature adolescents with GCTB that is unresectable or where surgical resection is likely to result in severe morbidity [95]. In Europe, the European Medicines Agency also approved it for GCTB in September 2014. In addition, denosumab is indicated for the prevention of skeletal-related events in patients with bone metastases from solid tumours in the US and in the European Union [94, 95].

The recommended dose of denosumab in the GCTB indication is 120 mg administered once every 4 weeks with additional 120 mg doses on days 8 and 15 of the first month of therapy. Denosumab is administered as a single subcutaneous injection in the upper arm, upper thigh, or abdomen [94, 95].

##### Mechanism of action

Denosumab is a human monoclonal antibody [immunoglobulin G2 (IgG2)] that targets and binds RANKL with high affinity and specificity, preventing activation of its receptor, RANK, on the surface of giant cells, osteoclast precursors and osteoclasts. Prevention of the RANK/RANKL interaction inhibits osteoclast formation, function, and survival, thereby decreasing bone resorption in GCTB (Fig. 1b) [96].

##### Pharmacokinetic and pharmacodynamic properties

Following subcutaneous administration, rapid and prolonged absorption of denosumab has been shown [96, 97]. It has been detected in the serum within 1 h of dosing and for up to 9 months following a single dose (maximal serum concentrations achieved between 5 and 21 days) [96, 97]. With multiple dosing (120 mg subcutaneously, every 4 weeks), there was an approximately twofold increase in serum concentrations in treated patients with bone metastases secondary to solid tumours. Steady state is attained by 6 months and, at steady state, the mean serum trough concentration is 20.5 μg/mL (standard deviation 13.5 μg/mL) and mean elimination half-life is 28 days [94].

##### Clinical development in GCT

The safety and efficacy of denosumab for the treatment of GCTB in adults or skeletally mature adolescents were demonstrated in two phase 2, open-label studies. All patients received 120 mg of denosumab subcutaneously every 4 weeks with additional doses on days 8 and 15 of the first cycle of therapy [98, 99].

*Efficacy* A single-arm, open-label, pharmacodynamic and proof of concept study evaluated the safety and efficacy of denosumab in 37 patients ≥18 years with recurrent or unresectable GCTB [99].

Eighty-six percent (95 % CI 70–95) of patients (*n* = 30) met the criteria for tumour response (elimination of ≥90 % of giant cells or no radiological progression of the target lesion): 20 based on histology and 10 based on radiology. Histological results showed near-complete or complete elimination of giant cells in all patients for whom histology was available. Improvement in functional status or reduced pain were reported in 84 % of patients (95 % CI 66–95; *n* = 26), and 29 % of patients (95 % CI 14–48; *n* = 9) had evidence of bone repair [99].

The second study was an open-label, single-arm, parallel-cohort, proof of concept, and safety trial conducted in 282 adult patients with primary or recurrent GCTB distributed in 3 cohorts [98]: cohort 1, 170 patients who had surgically unsalvageable disease as determined by the treating surgeon (e.g. sacral, spinal, or multiple GCTB lesions including pulmonary metastases); cohort 2, 101 patients with a planned surgery that was associated with severe morbidity (e.g. joint resection, limb amputation, hemipelvectomy); and cohort 3, 11 patients who transitioned from the previous denosumab GCTB study [99] and continued denosumab treatment on this study. The primary efficacy outcome measures were time to disease progression in cohort 1 and the proportion of patients without any surgery at 6 months in cohort 2 [98]. An interim analysis was published when more than 200 patients had had an opportunity to complete 6 months of treatment after enrolment [98].

At the time of the interim analysis, median time to disease progression in cohort 1 was not reached, and the best response rate (complete or partial) determined by investigator was 41 % in cohort 1 and 58 % in cohort 2 (Table 2). In a retrospective, independent imaging analysis that evaluated tumour response in patients from all three cohorts who received imaging as part of their standard of care (*N* = 190), the overall objective response rate (RECIST 1.1) was 25 % (95 % CI 19, 32), with all responses documented as partial responses. The estimated median time to response was 3 months. In the 47 patients with an objective response, median duration of follow-up was 20 months (range 2–44 months), and 51 % (24/47) had a duration of response lasting at least 8 months. Three patients experienced disease progression following response. Combining three different response criteria (RECIST, European Organization for Research and Treatment of Cancer (EORTC) and Modified Choi criteria), the best objective response rate was 72 % (Table 2) [98].

**Table 2:** Main results of the phase 2 study of denosumab in GCTB [98]

	Best response (investigator-determined)			
	Cohort 1: surgically unsalvageable		Cohort 2: salvageable, surgery planned	
Complete response, % (*n*/*N*1)	5 (8/159)		18 (17/93)	
Partial response, % (*n*/*N*1)	36 (57/159)		40 (37/93)	
Stable disease, % (*n*/*N*1)	58 (93/159)		41 (38/93)	
Disease progression, % (*n*/*N*1)	1 (1/159)		1 (1/93)	

	Best clinical benefit (investigator-determined)			
Pain reduction, % (*n*/*N*)	28 (48/169)		50 (50/100)	
Improved mobility, % (*n*/*N*)	22 (38/169)		33 (33/100)	
Improved function, % (*n*/*N*)	19 (32/169)		23 (23/100)	
Other, % (*n*/*N*)	4 (6/169)		10 (10/100)	

	Best response (independent imaging assessment)			
	Overall	RECIST 1.1	EORTC	Inverse Choi
Objective response (OR)^a^, % (*n*/*N*2)	72 (136/190)	25 (47/187)	96 (25/26)	76 (134/176)
Median time to OR, months	3.1	not reached	2.7	3
OR sustained ≥24 weeks, % (*n*/*N*2*)	68 (76/111)	24 (26/109)	92 (11/12)	75 (76/102)
Tumour control^b^ sustained ≥24 weeks, % (*n*/*N*2*)	98 (109/111)	99.1 (108/109)	100 (12/12)	99 (101/102)

*N*1 number of enrolled patients who received ≥1 dose of denosumab and had a disease status evaluation

*N* number of enrolled subjects who were eligible for the study and received ≥1 dose of denosumab

*N*2 Patients with ≥1 evaluable timepoint assessment

*RECIST* response evaluation criteria in solid tumours, *EORTC* European organization for research and treatment of cancer

*Patients with timepoint assessments ≥24 weeks apart

^a^Objective response = complete + partial response

^b^Tumour control = complete + partial response + stable disease

Clinical benefit was observed in 40 and 61 % of patients in cohorts 1 and 2, respectively, with pain reduction the most commonly observed benefit (Table 2; Fig. 2). Of the 100 patients in cohort 2 for whom surgery was planned at baseline, 90 (90 %) patients had either no surgery (*n* = 74; 74 %) or underwent a less morbid procedure (*n* = 16; 16 %) compared with the surgical procedure planned at baseline [98] (Table 3; Fig. 2). Median follow-up for cohort 2 was 9.2 months (IQR 4.2–12.9). Of the 71 patients who were on study for at least 6 months, 64 (90 %) did not have surgery by month 6. Of the 26 patients who had surgery, the median time to surgery was 23.8 months.

**Table 3:** Planned versus actual surgeries in cohort 2 of the phase 2 study of denosumab in GCTB [98]

	Planned	Actual total
Surgical procedure, *n* ^a^	(*N* = 100)	(*N* = 26)
Total number of surgeries	100	26
Major surgeries	44	3
Hemipelvectomy	4	0
Amputation	17	0
Joint or prosthesis replacement	9	1
Joint resection	14	2
En bloc resection	37	6
En bloc excision	4	0
Marginal excision	1	0
Curettage	13	16
Other	1	1
No surgery	NA	74

*NA* not applicable

^a^Data are *n* in the efficacy analysis set. Procedures are in decreasing order of morbidity

*Safety* In the first phase 2 study, 89 % of patients experienced an adverse event (AE) with the most frequently reported AEs being pain in the extremity, back pain, and headache. One case of osteonecrosis of the jaw (ONJ) was also reported [100].

In the second phase 2 study, 84 % of patients who received at least one dose of denosumab reported an AE. Commonly reported AEs included arthralgia, headache, nausea, and fatigue. The incidence of hypercalcemia was 5 %, none of which were judged to be serious, and the incidence of ONJ was 1 % (3 patients) [98].

During treatment with denosumab, it is recommended that calcium levels should be monitored, and all patients should receive daily calcium and vitamin D supplementation. A dental examination with appropriate preventive dentistry should be considered before initiating treatment with denosumab and invasive dental procedures should be avoided during the course of treatment. Oral examinations should be performed regularly by both the patient and physician [94, 95].

### Other studies

A case series also suggested that preoperative treatment with denosumab induces dramatic sclerosis and reconstitution of cortical bone, achieving tumour necrosis in 90 % of patients. The authors reported that, after denosumab treatment, subsequent surgical resection was easier in cases of aggressive tumours and that denosumab should also be considered as a stand-alone treatment in patients who are poor surgical candidates or in cases where the tumour is in a location difficult to treat surgically [101]. There are also some case reports of successful use of denosumab in children [102], although it has not been formally assessed in this population and is not recommended for use.

## IFN-α/PEG-IFN

The increased expression of several angiogenic growth factors observed in GCTB led to the use of interferon alfa (IFN-α) as an anti-angiogenic agent. The first use was in 1995 [103], and since then several studies have reported successful treatment of GCTB with this agent [104]. Pegylated (PEG)-IFN has also been shown to have anti-GCTB activity. A few case reports have reported the efficacy of interferon and pegylated interferon in the management of GCTB [105].

### Bisphosphonates

Due to their anti-resorptive properties, some exploratory studies tested the efficacy of bisphosphonates in GCTB. It was shown that nitrogen-containing bisphosphonates induce apoptosis in both giant cells and stromal cells in vitro [106]. In a case–control study, pamidronate and zoledronate reduced local tumour recurrence (4.2 vs 30 % in the control group, *p* = 0.056) and controlled disease progression when used orally or intravenously as adjuvant therapy to intralesional curettage [107]. In 25 patients with recurrent and metastatic GCTB treated with bisphosphonates, stabilisation of disease was achieved in most cases refractory to conventional treatment [108]. In addition, there are case reports of successful local administration of zoledronic acid as adjuvant therapy during surgery [109]. However, they are not approved for use in this indication and more evidence is needed.

### Current guideline recommendations

#### NCCN

In 2013, the National Comprehensive Cancer Network (NCCN) Clinical Practice Guidelines in Oncology for bone cancer added a new section on GCTB.

According to the version 1.2015 of these guidelines, workup begins with a history, physical examination, cross-sectional imaging of the primary site, chest imaging, and biopsy to confirm the diagnosis. Bone scan is considered optional [110].

Regarding treatment (Table 4), the decision tree depends on whether the disease is localised or metastatic. For localised disease, the choice of surgery is next. If the tumour is resectable, excision is the primary option. If the tumour is resectable with unacceptable morbidity or unresectable, the options include serial embolization (primarily for tumours of the pelvis), denosumab, interferon, pegylated interferon, and/or radiotherapy [110].

**Table 4:** 2015 NCCN recommendations for GCTB [110]

Giant cell tumour of the bone—NCCN guidelines (Version 1.2015)		
	Treatment	Follow-up
Localised disease (primary or recurrent)		
Resectable	Excision (in recurrence: consider chest imaging and/or denosumab prior to surgery)	Physical exam
		Imaging of surgical site as clinically indicated
		Chest imaging every 6 m for 2 years then annually
Resectable with unacceptable morbidity * and/or* Unresectable	Serial embolization *and/or* Denosumab *and/or* IFN or PEG-IFN *and/or* RT	*If stable/improved disease* Same follow-up as after excision *If stable/improved disease with incomplete healing* Excision (if resectable) Continue on-treatment (if unresectable) *If progressive disease* Continue on-treatment
Metastatic disease (at presentation or recurrence)		
Resectable	Treat primary tumour Consider excision of metastasis	Physical exam Imaging of surgical site as clinically indicated Chest imaging every 6 m for 2 years then annually
Unresectable	Denosumab *and/or* IFN or PEG-IFN and/or RT *and/or* Observation	*If stable/improved disease* Same follow-up as after excision *If stable/improved disease with incomplete healing* Excision (if resectable) Continue on-treatment (if unresectable) *If progressive disease* Continue on-treatment

*IFN* interferon, *NCCN* national comprehensive cancer network, *PEG* pegylated, *RT* radiotherapy

For metastatic disease, the feasibility of surgery determines the treatment options. If the tumour is resectable, again the primary treatment pathway for localised disease should be followed and excision of metastatic sites considered. If the tumour is unresectable, treatment options include denosumab, interferon, pegylated interferon, radiotherapy, or observation [110].

NCCN Guidelines also contain recommendations for surveillance, which include physical examination, imaging of the surgical site as clinically indicated, and chest imaging every 6 months for 2 years and annually thereafter. For a resectable local tumour recurrence, chest imaging and denosumab may be considered before surgery [110].

#### ESMO

The 2014 ESMO guidelines for bone sarcomas [111] specify that treatment options for GCTB include intralesional curettage with or without adjuvant or en bloc excision. They also mention that recent work has suggested that denosumab obtains substantial tumour responses in large or unresectable or metastatic GCTB. For this reason, denosumab may be used to achieve cytoreduction allowing potentially curative surgery, or also in unresectable and rare metastatic disease, where treatment needs to be maintained to avoid progression [111].

Regarding surveillance, the recommendation for low-grade bone sarcomas such as GCTB, include follow-up visits every 6 months for 2 years and then annually. However, they comment that late metastases as well as local recurrences and functional deficits may occur >10 years after diagnosis and that there is no universally accepted stopping point for tumour surveillance [111].

### Future expectations

The knowledge of GCTB pathophysiology is rapidly evolving. The identification of the chemotactic factors secreted by stromal cells and involved in monocyte transformation into giant cells provides an opportunity to discover innovative treatments. The monoclonal antibody denosumab is the first drug agent with proven efficacy in GCTB by targeting one of these factors (RANKL). The main pending questions with denosumab include the evaluation of its possible benefits as neoadjuvant therapy [112], the optimal duration and schedule of treatment at long term to avoid recurrences, and its long-term safety. Some angiogenesis inhibitors have also been tested, such as calcitonin and interferon. IFN-α inhibits the expression of b-FGF and IL-8, two angiogenic factors. Other candidate therapies could be monoclonal antibodies directed against the involved cytokines or enzymes, such as anti-IL6, cathepsin inhibitors, anti-M-CSF or MMP-specific inhibitors [113]. The newer antibody–drug conjugates (ADCs), a novel class of highly potent drugs composed of an antibody (a whole antibody or an antibody fragment) linked to a cytotoxic drug could revolutionise treatment of GCTB [114]. Although few ADCs are currently available [115], there are more than 20 compounds currently in clinical development, specific for a wide range of biological targets expressed by tumour cells [116]. It is hoped that, in the near future, some of them could be suitable for GCTB, in view of promising results in other cancers.

It also seems that targeting the neoplastic stromal cells could fight directly against the origin of tumour. Therapies blocking proliferation of stromal cells, such as drugs inhibiting cell cycle progression or telomerase activity could be effective. First, it would be necessary to identify specific markers for the stromal cells.

Recent findings suggest that the haemorrhagic component plays a fundamental role in the development of giant cells. In some instances, GCTB could be a reactive condition secondary to massive intraosseous haemorrhage, which attracts monocytes and forces their quick proliferation and conversion into multinucleated cells. There is also the hypothesis that poor matrix support to the vessels may underlie the haemorrhage that precedes tumour formation. Currently, the use of embolisation techniques and occlusion of the vessels helps reduce recurrence. Other treatments aimed to occlude the vessels and reinforce local osseous matrix support, such as laser and hormone therapies, could be also effective.

A more deep investigation on genetic predisposition may help to identify individuals at higher recurrence risk, in whom more aggressive therapies should be undertaken. For example, amplification of 20q11.1 seems to be a prognostic marker for adverse outcome [117] and warrants further investigation.

## Conclusions

GCTB is an aggressive primary osteolytic bone tumour that causes substantial morbidity. GCTB tumours contain osteoclast-like giant cells that express RANK and stromal cells that express RANKL, a key mediator of osteoclast formation, activation, function, and survival. Excessive secretion of RANKL causes an imbalance in bone remodelling in favour of bone breakdown. Before the discovery of denosumab, surgical intervention was the only definitive therapy for patients with resectable tumours; however, it is associated with significant morbidity. Currently, denosumab constitutes an effective therapeutic option for treatment of adult patients with unresectable GCTB or in whom surgical resection is likely to result in severe morbidity. Denosumab provides objective tumour responses in 72 % of patients, prolonging the time to surgery and reducing its morbidity in those patients with planned interventions. Denosumab is well tolerated, with ONJ and hypocalcemia; known risks are observed at low rates. The increasing knowledge of the molecular mechanisms involved in GCTB pathophysiology provides an opportunity for using new targeted therapies that may dramatically change the outcomes of GCTB in the next years.
